# Pyridoxal 5′-Phosphate Biosynthesis by Pyridox-(am)-ine 5′-Phosphate Oxidase: Species-Specific Features

**DOI:** 10.3390/ijms25063174

**Published:** 2024-03-09

**Authors:** Maribel Rivero, Nerea Novo, Milagros Medina

**Affiliations:** 1 Department of Biochemistry and Molecular and Cellular Biology, Faculty of Sciences, University of Zaragoza, 50009 Zaragoza, Spain; miriverob01@gmail.com (M.R.); nerea.nnh@gmail.com (N.N.); 2 Institute for Biocomputation and Physics of Complex Systems (BIFI), University of Zaragoza, 50018 Zaragoza, Spain

**Keywords:** pyridox-(am)-ine 5′-phosphate oxidase (PNPOx), pyridoxal 5′-phosphate (PLP), flavin mononucleotide, species-specific features, conformational landscape, cofactor channelling

## Abstract

Enzymes reliant on pyridoxal 5′-phosphate (PLP), the metabolically active form of vitamin B_6_, hold significant importance in both biology and medicine. They facilitate various biochemical reactions, particularly in amino acid and neurotransmitter metabolisms. Vitamin B_6_ is absorbed by organisms in its non-phosphorylated form and phosphorylated within cells via pyridoxal kinase (PLK) and pyridox-(am)-ine 5′-phosphate oxidase (PNPOx). The flavin mononucleotide-dependent PNPOx enzyme converts pyridoxine 5′-phosphate and pyridoxamine 5′-phosphate into PLP. PNPOx is vital for both biosynthesis and salvage pathways in organisms producing B_6_ vitamers. However, for those depending on vitamin B_6_ as a nutrient, PNPOx participates only in the salvage pathway. Transferring the PLP produced via PNPOx to client apo-enzymes is indispensable for their catalytic function, proper folding and targeting of specific organelles. PNPOx activity deficiencies due to inborn errors lead to severe neurological pathologies, particularly neonatal epileptic encephalopathy. PNPOx maintains PLP homeostasis through highly regulated mechanisms, including structural alterations throughout the catalytic cycle and allosteric PLP binding, influencing substrate transformation at the active site. Elucidation at the molecular level of the mechanisms underlying PNPOx activity deficiencies is a requirement to develop personalized approaches to treat related disorders. Finally, despite shared features, the few PNPOx enzymes molecularly and functionally studied show species-specific regulatory properties that open the possibility of targeting it in pathogenic organisms.

## 1. Vitamin B_6_: Vitamers and Metabolism

**Figure 1:**
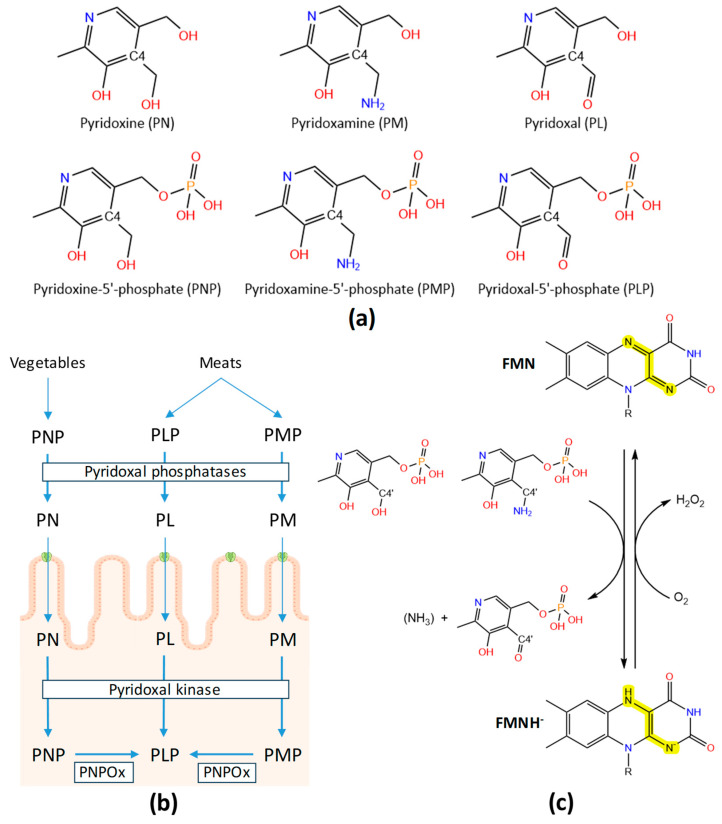
Vitamin B_6_. (**a**) Molecular structures of the B_6_ vitamers. (**b**) Overview of PLP metabolism. (**c**) Flavin reductive and oxidative half-reactions during the catalytic cycle of PNPOx.

Vitamins B form a group of six hydro-soluble molecules that are essential nutrients in cell bioenergetics and metabolism. This group includes thiamine (vitamin B_1_), riboflavin (vitamin B_2_), niacin (vitamin B_3_), pantothenic acid (vitamin B_5_), pyridoxine (vitamin B_6_), biotin (vitamin B_7_), folic acid (vitamin B_9_) and cobalamin (vitamin B_12_). In the case of vitamin B_6_, this is the generic name for up to six different vitamers that confer final vitamin B_6_ activity: pyridoxine (PN), pyridoxal (PL), and pyridoxamine (PM), as well as their respective 5′-phosphate esters, pyridoxamine-5′-phosphate (PNP), pyridoxal-5′-phosphate (PLP) and pyridoxamine-5′-phosphate (PMP) (Figure 1a, Appendix A). Chemically, B_6_ vitamers are derivatives of 2-methyl-3-hydroxy-5-hydroxymethyl-pyridine while their C4 substituent being hydroxymethyl (-CH_2_OH), aminomethyl (-CH_2_NH_2_) and aldehyde (-CHO) groups in PN, PM and PL, respectively. B_6_ vitamers are water-soluble and naturally present in the diet in many forms and foods, added to others, and available as a dietary supplement. Nonetheless, substantial proportions of the naturally occurring PN in fruits, vegetables and grains exist in glycosylated forms that exhibit reduced bioavailability. The biologically active form of vitamin B_6_ is PLP, functioning as a cofactor in nearly 200 PLP-dependent activities carried out via enzymes, which account for up to 4% of enzyme-catalysed reactions [1,2] (Appendix B). Such reactions include those within amino acid and glycogen metabolism, the synthesis of nucleic acids, hemoglobin and sphingomyelin, as well as the biosynthesis and degradation of multiple neurotransmitters [3,4,5]. PLP is also involved in the metabolism of one-carbon units, carbohydrates and lipids, and changes in its physiological concentration contribute to the circadian control of enzyme activities in the brain and peripheral tissues [6].

De novo pathways for PLP biosynthesis are active only in prokaryotes and plants, in which PNP, for example, is synthesised from deoxy-xylulose 5-phosphate and 4-phosphohydroxy-L-threonine in *Escherichia coli* [7,8], or from intermediates from the pentose phosphate pathway and glycolysis in other bacteria and plants [9]. In contrast, the salvage pathway is involved in the interconversion between different B_6_ vitamers, requiring the action of pyridoxal kinase (PLK), pyridox(-am-)ine 5′-phosphate oxidase (PNPOx) and phosphatases, and is active in both prokaryotes and eukaryotes. Therefore, humans must be supplied with this vitamin. The main sources of B_6_ vitamers for humans are the diet, the degradation of PLP-dependent enzymes and the action of the gut microbiota, with the latter making a significant contribution [10]. Dietary deficiency of vitamin B_6_ is generally rare but can occur in the first year of life when the gut flora is not fully established, as well as in patients with chronic alcoholism, diabetes mellitus, coeliac disease and those on long-term use of isoniazid or penicillamine therapeutic agents [1,11]. On the contrary, PNPOx levels significantly increase in some hemodialysis patients, which is being investigated as a potential causative predisposition to infection [12]. The human body only absorbs the non-phosphorylated B_6_ vitamers in a process that occurs in the jejunum portion of the intestine [1,2]. For that, phosphorylated B_6_ vitamers are dephosphorylated via intestinal phosphatases and a glycosidase (Figure 1b). The absorption of the pool of non-phosphorylated vitamin B_6_ is suggested to occur via passive diffusion across the cell membrane, attached to amino acids/peptides or transported as a sugar adduct, but vitamin B_6_ transporters are still to be identified in all types of organisms [10,13,14]. Some studies envisage the existence of PN-specific and regulated carrier-mediated processes [11,15,16]. Such a transport process would be temperature-, energy-, and pH-dependent, suggesting it may occur via a PN:H^+^ symporter mechanism [15,17]. In addition, it appears to be under the regulation of an intracellular protein kinase A-mediated pathway. More recent studies have also shown that some transporters involved in the uptake of other water-soluble vitamins can also contribute to PN transport, including SLC19A and SLC19A3 [18]. Nonetheless, such possibility of PN transport appears species-specific, and so far, no specific transport protein of any mammalian species has been characterized at the molecular level. In *Saccharomyces cerevisiae*, Tpn1p was identified as the plasma membrane vitamin B_6_ transporter, with broad substrate specificity for unphosphorylated B_6_ vitamers, utilizing a proton symporter mechanism and with residues at its 4-transmembrane segment being key for functionality [19]. Therefore, the roles of different transporters in vitamin B_6_ metabolism and their individual contributions to the uptake of PN in different species are areas of ongoing research. PN and PM can be converted to PLP in the intestine cells prior to transport to the liver or once within it. PM, PN and PL are first re-phosphorylated via PLK, and then, the FMN-dependent PNPOx transforms PMP and PNP into PLP, reducing molecular oxygen to hydrogen peroxide (Figure 1b). Although this occurs mainly in the liver, PNPOx is expressed in all cell types in Eukarya as well as in all types of Bacteria [1]. PLP is exported from the liver bound to albumin, but to enter the brain, it must dissociate and be again dephosphorylated to PL at the blood–brain barrier. Within tissues such as the brain, partial catalysis via PLP or catabolism of PLP enzymes can also lead to the production of PMP, which can be converted back to PLP via PNPOx through the salvage pathway.

The versatility of PLP arises from its ability to covalently bind to its client enzymatic substrates and then act as an electrophilic catalyst, thereby stabilizing different types of carbanionic reaction intermediates. Within client enzymes, the reactive aldehyde group of PLP generally undergoes a condensation reaction with the amino group of amino acids to produce a Schiff base. In humans, PLP-dependent holoenzymes contain PLP attached via a Schiff base linked to the ε-amino group of a lysine residue at the active site. Moreover, most reactions involve the transfer of PLP to produce a new Schiff base linkage with the amino group of an amino acid at the substrate. B_6_ vitamers may also play other roles within the cell, such as antioxidants, modifying the expression and action of steroid hormone receptors and impacting immune function [20]. PLP has also been reported to exhibit anti-epileptic activity because it is an antagonist of ATP at the P2 pyrinoceptor [1]. Nonetheless, PLP is a very reactive molecule and can be very toxic, causing toxicity in the liver and being involved in unwanted reactions [21]. To avoid such processes, the cells maintain intracellular levels of free PLP at approximately 1 µM [1]. Several mechanisms contribute to maintaining such low PLP concentrations in cells and body fluids: (i) PLP-dependent enzymes that keep it inbound state, (ii) inhibition via PLP of PLK and PNPOx, and (iii) PLP degradation via phosphatases. In addition, in vitro studies suggest that PLP will be protected intracellularly by being transferred directly from PLK and PNPOx to some of the PLP-dependent enzymes [22].

## 2. Functional and Structural Features of Human PNPOx

The human *PNPO (HsPNPO)* gene is located on chromosome 17 (17q21.2). It encodes a protein of 261 aa (~30 kDa, Q9NVS9 · PNPO_HUMAN) and exhibits housekeeping characteristics: the absence of TATA-like sequences and the presence of Sp1-binding sites and, more importantly, CpG islands in the regulatory region. Its mRNA expression is ubiquitous, but its transcription is highly regulated in a tissue-specific manner. Its major sites of expression are the liver, skeletal muscle and kidneys, but it is also expressed in the heart, brain and pancreas [23]. The cDNA for *HsPNPO* was cloned and expressed in *E. coli*, where it was efficiently overproduced and purified [24]. Once produced, the HsPNPOx protein incorporates FMN as a cofactor, which enables it to catalyse the conversion of both PNP and PMP to the biologically active PLP (Figure 1b). Thus, HsPNPOx is able to oxidize both primary alcohol and primary amino groups to an aldehyde group [25]. Catalysis occurs through the initial transfer of a pair of electrons (as a hydride) from the C4′ of PNP (or PMP) to the tightly bound FMN, forming FMNH^−^ and PLP, during the so-called flavin semi-reductive half-reaction (Figure 1c). Then, the electron pair is transferred in the oxidative half-reaction from FMNH^−^ to O_2_, regenerating oxidized FMN and forming H_2_O_2_ [26]. HsPNPO was confirmed to be a homodimer that presents characteristic absorption maxima for a flavoprotein at 276 nm, 385 nm and 448 nm [27]. Noticeably, HsPNPOx exhibits considerably low *k*_cat_ as well as *K*_M_ for both PNP and PMP, with affinities for both substrates being in the low micromolar range (Table 1) [27]. Steady-state studies revealed a pre-steady-state transient period where the rate of PLP synthesis was slower than normal, thought to be consistent with the few seconds whilst enzyme and substrate form the complex. Although the FMN of HsPNPOx masking spectroscopic observation of tightly bound PLP, the strong binding of PLP to apo-HsPNPOx was spectroscopically confirmed (peak at ~410 nm). Thus, PLP was reported as an effective product/competitive inhibitor of HsPNPOx. However, the catalytic activity of preformed HsPNPOx:PLP complexes did not appear to be affected in vivo because *K*_i_^PLP^ was not low enough for it to be tightly bound at the active site. This indicated that tight binding of PLP occurred at an alternative non-catalytic site and suggested that a tunnel may exist between the active site and the secondary non-catalytic site [28]. This allosteric PLP binding site is key in enzyme regulation, produces allosteric inhibition, and has been proposed to act as protecting PLP from nucleophiles as well as channel it to client apo-enzymes. Nonetheless, in HsPNPOx, coupling between catalytic and allosteric sites appears to be weak, allowing the formation of complexes with PLP at the allosteric site and PNP at the catalytic site that still remain partially active [29]. Moreover, while free HsPNPOx shows its active site open, the formation of the HsPNPOx:PLP complex partially closes it because of the bound PLP blocking solvent access.

**Figure 2:**
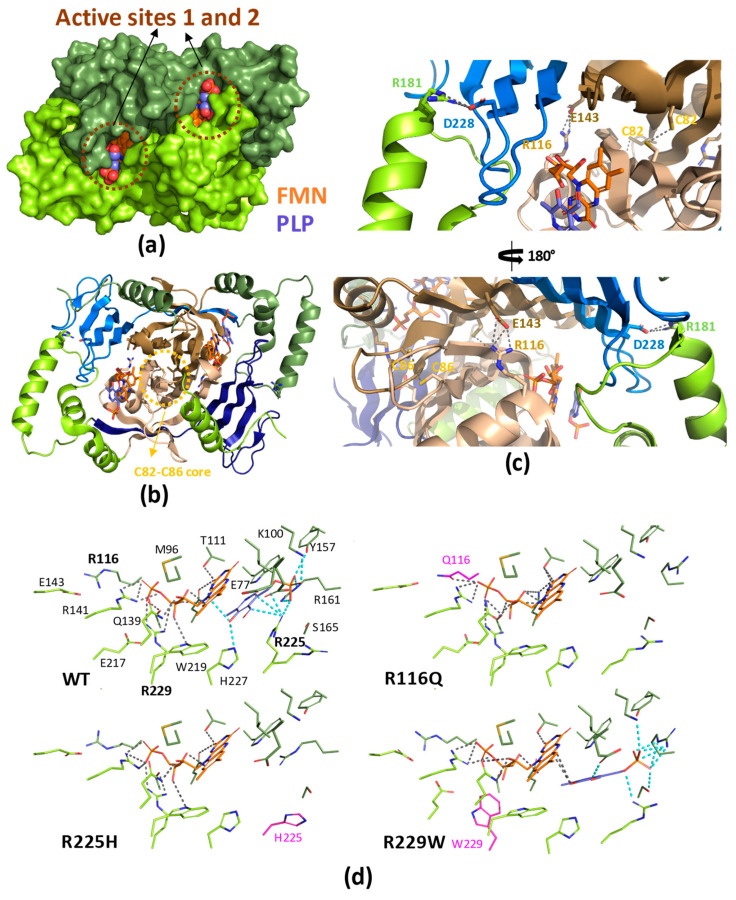
Structural features of HsPNPOx. (**a**) Surface representation of the crystallographic structure of WT HsPNPOx homodimer in complex with PLP at the active site (PDB 1NRG). (**b**) Cartoon model of the homodimer, with the Putative_PNPOx domains in each protomer, highlighted in dark and light sand, respectively, and the corresponding PNP_phzG_C domains in light and dark blue. Residues involved in potential inter-subunit contacts are highlighted in the sticks. (**c**) Detail of inter-subunit contacts. The top and bottom panels correspond to the view centred at the FMN of one active site upon 180° rotation. (**d**) Detail of the active site environment in WT in the complex with PLP, the R116Q mutant (6H00), the R225H mutant (8QYW) and the R229W mutant in the complex with PLP (3HY8). Mutated residues are highlighted in magenta. Polar contacts for FMN and PLP are shown as dashed lines in grey and cyan, respectively. Other colour codes as in panel (**a**). In all panels, one protomer of the homodimer is shown in smudge green and the other in lemon green, while FMN and PLP are shown in CPK, coloured in orange and violet spheres or sticks. Figure produced using PyMOL, respectively [39].

**Figure 3:**
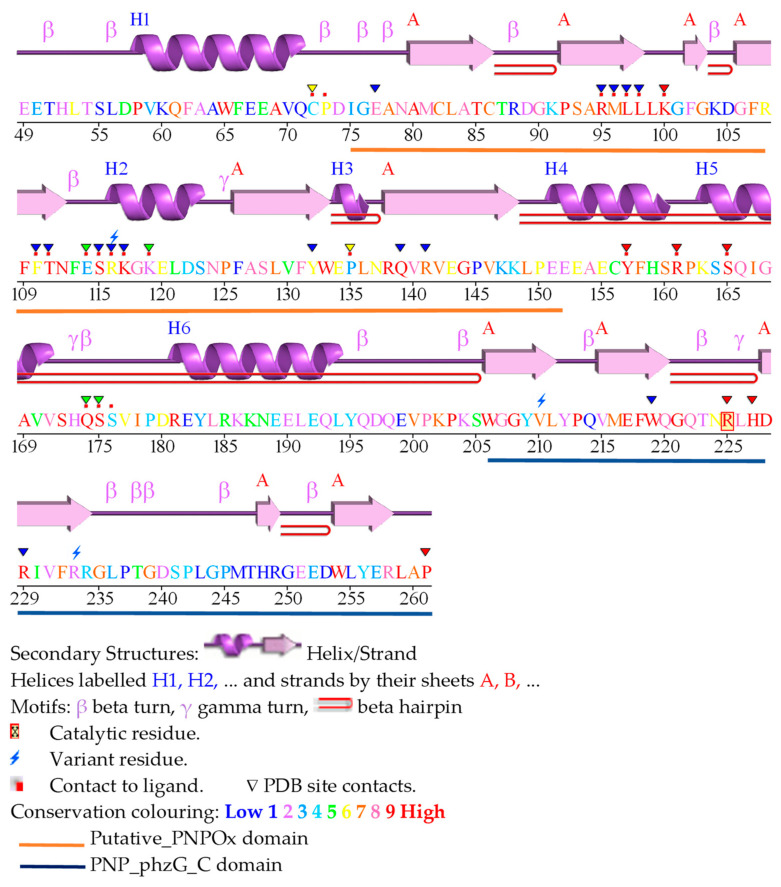
PDBsum analysis of HsPNPOx. Secondary structure analysis obtained from the PDBsum server (https://www.ebi.ac.uk/thornton-srv/databases/pdbsum/) for PDB 1NRG (Accessed on 15th January 2024).

**Figure 4:**
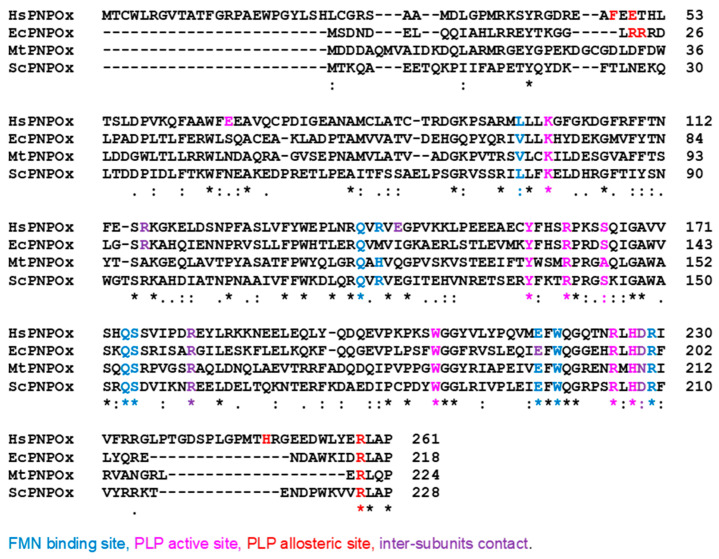
Multiple sequence alignment (MSA) (Clustal Omega) of WT PNPOx sequences from species with available crystallographic structures. Asterisk (*), colon (:) and period (.) symbols indicate identical residues, conserved substitutions and semi-conserved substitutions, respectively. Colours highlight residues involved in the binding of particular ligands or inter-subunit binding.

The PDB contains five structures for HsPNPOx; two of the wild-type (WT, PDB ID 1NRG and 8QYT) and three corresponding to the mutants, R229W (3HY8), R116Q (6H00) and R225H (8QYW) [24,27,30]. The structures for the WT and R229W variants are complexes with PLP, showing it in close proximity to the FMN cofactor at the active site. On the other hand, the structures of the R116Q and R225H variants correspond to the PLP-free protein. In general, they do not show the first 48–50 N-terminal residues because of their high predicted disorder. The functional HsPNPOx structure is a homodimer with an FMN and a substrate binding site in each monomer (Figure 2a,b). Each HsPNPOx monomer exhibits a two-domain architecture represented by two PFAM domains [27]. The larger domain, a Putative_PNPOx domain (PFAM: PF01243, IPRO11576, residues 75–153), is formed using six β-strands and two short α-helix, while the smaller domain, a PNP_phzG_C domain, is made up of five β-strands (PFAM: PF10590.9, IPRO19576, 206–261). In addition, one additional α-helix precedes the Putative_PNPOx domain, and three more make the inter-domain link (residues 154–205) between PFAM domains (Figure 2b and Figure 3). Several of the β-strands organize in β-hairpins, and all of them make a β-sheet. The functional dimer is stabilized via salt-bridge interactions between the two monomers, particularly Arg116-Glu143 and Arg181-Asp228. The enzyme contains up to six Cys residues per monomer that apparently are not involved in catalytic activity, although two of them, Cys82 and Cys86, might be implicated in Cys82-Cys82 and Cys86-Cys86 disulphide bonds formed at the interface between both monomers (Figure 2b,c). FMN is located in a deep cleft formed through the two protein domains, interacting with both monomers (both via H-bond and water-mediated links) and contributing to inter-subunit interactions (Figure 2a–c). These interactions stabilize FMN binding and may also ensure the correct isoalloxazine orientation for the catalytic activity. Residues participating in these interactions are Thr111, Gln139, Arg141, Gln174, Ser175, Glu217, Trp219 and Arg229, with the latter making an ion-pair with the phosphate of FMN (Figure 2c) [27]. Additionally, both protein domains and monomers are involved in PLP binding at the active site. The WT HsPNPOx:PLP structure shows the C4′ of PLP situated in front of N5 at the re-face of FMN, with the phosphate group pointing out of the cavity mouth and interacting with residues from both monomers: Lys100A, Tyr157A, Arg161A, Ser165A and Arg225B (Figure 2c). The pyridine ring stacks against the isoalloxazine of FMN and is allocated within residues of both chains in the homodimer: Glu77A, Trp206A, Arg225B and His227B. In particular, Arg225 and His227 orientate substrate binding for catalysis, placing the substrate pyridine ring parallel to the FMN isoalloxazine ring so the distance between the C4′ of PNP and the N5 of FMN would allow efficient hydride transfer [24]. With the only exception of Glu77, these residues are highly conserved in other species (Figure 3 and Figure 4).

As indicated above, the R225H PNPOx variant is one of the few for which the crystal structure is available (PDB 8QYW). The mutation hardly has any impact on the overall fold (RMSD ~0.3 Å) and active site architecture of the protein, but this replacement will prevent the correct coordination of the phosphate of PLP and the correct allocation of the substrate for catalysis (Figure 2d). The replacement of Arg229 with a Trp is also among the best biochemically characterized mutations of HsPNPOx (PDB 3HY8). The R229W mutant and WT structures show identical overall folds (RMSD ~0.6 Å), but the mutation leads to the loss of the interaction of residue 229 with the phosphate of FMN as well as a change in the electrostatics for FMN binding. The substituted W229 is too big to be accommodated in the FMN binding pocket, inducing a movement in the loop containing His227 and Arg225 to relieve steric interaction and resulting in the loss of two other critical H-bonds (Figure 2d). The structural perturbation caused by the R229W mutation affects not only the FMN phosphate binding site but also the substrate binding site because it removes critical H-bond interactions of the substrate with His227 and Arg225. Therefore, the orientation of the substrate is apparently altered, with the PLP and FMN ring moieties no longer parallel (Figure 2d). As will be discussed below, this mutation impacts the affinities of FMN and PNP, as well as the hydride transfer efficiency [24]. The other pathogenic variant for which a crystal structure is available is R116Q. Arg116 is located at the N-terminal end of the α-helix, whose positive dipole charge might stabilize the negative charge of the FMN phosphate. In addition, it makes an ion-pair interaction with the neighbouring Glu143 residue from the other protomer (Figure 2d). No major differences are found in the R116Q mutant’s overall structure when compared to the native protein. However, the Gln side chain, being shorter than that of the Arg, leaves the introduced amide at the bonding distance of the FMN phosphate but not of Glu143. Therefore, in solution, this replacement might have a direct impact on FMN affinity, homodimer stability and/or inter-subunit flexibility.

## 3. The Human PNPOx Enzyme in Disease

The binding of the PLP product of HsPNPOx to its client apo-enzymes is essential for their catalytic function and, in many cases, also for their correct folding and targeting to the right organelle [22]. Moreover, considering the large number of reactions relying on PLP, including its vital role in neurotransmitter metabolism (synthesis of inhibitory transmitter GABA), severe neurological disorders, such as convulsions and epileptic encephalopathy, result from reduced availability of PLP in the cell [40,41]. Such deficiency is, in many cases, due to inborn errors in the enzymes of the salvage pathway. In particular, in patients suffering from PNPOx deficiency (PNPOD, OMIM #610090), PLP cannot be synthesised from dietary PN (present in vegetables and added as a B_6_ supplement to infant formulae and parenteral nutrition), nor can it be regenerated via recycling of PMP. Due to its key role in brain metabolism, the mutation or deficiency of this enzyme in humans is linked to neurological pathologies, with neonatal epileptic encephalopathy being the most remarkable one [42,43]. PNPOD is an autosomal recessive inborn error of metabolism that leads to a seizure disorder, presenting in the new-born period or early infancy [44]. Clinical phenotypes and other general features observed include foetus distress, hypoglycaemia, acidosis, anaemia and asphyxia [24]. Progressive deterioration can lead to death within weeks. Cerebrospinal fluid and urine analyses indicate reduced activities of PLP-dependent enzymes in PNPOD. Under such situations, surviving children are usually mentally retarded and dependent on vitamin B_6_ in the form of PLP [24]. Moreover, genome-wide association studies identified an important susceptibility locus for genetic generalized epilepsies at 17q21.32 (rs72823592), the closest gene to which is *HsPNPO*, suggesting that mild PNPOx deficiency could be a susceptibility factor for genetic generalized epilepsies presenting at various ages [44]. PNPOD is a potentially treatable disease that, for many years, has been underdiagnosed or misdiagnosed due to the lack of specific biomarkers [45,46,47]. Treatment with PLP has been shown to be very effective in controlling seizures in PNPOD, with some patients even showing normal development and the treatment being lifelong [48,49]. On the contrary, with late or no treatment, patients may die or show severe mental handicaps. Some PNPOD seizures were observed to respond to treatment with PLP but not to PN [44]; nonetheless, some patients’ seizures also responded to PN treatment [50], and others responded to PN but not to PLP [46,51]. Different susceptibilities of patients to PLP or PN treatments, as well as individuals showing severe to mild phenotypes, are explained because PNPOD can be caused by different point and non-sense mutations as well as deletions/insertions and frame shifts in the *HsPNPO* gene (see Table 3 in [42]). For these reasons, the treatment of PNPOD has been relatively complicated. Some mutations (such as R225H) have, in fact, been reported to be responsive to PN but not to PLP, which seemed to inhibit the mutant’s activity instead. Others (R225H/C and D33V) result in seizures more likely to be responsive to PN, while some (R116Q) dramatically affect erythrocyte PNPOx activity. In general, mutations affecting PLP binding can be treated with PN (but worsen after treatment with PLP), while mutations affecting FMN binding can only be treated with PLP. In addition, the riboflavin status, which might work as a pharmacological chaperon in selected PNPOx variants failing FMN binding or the adequate supply of vitamin B_6_ to the foetus by the mother, has been pointed out as a factor positively influencing treatment response [1,44].

Naturally occurring HsPNPOx variants have been separated into categories based on their position in the protein structure and type of mutation (see Figure 3 and Table 3 in [42]). The most populated category contains mutations that directly affect the catalytic site and impair coordination via positively charged residues of phosphates of PLP and FMN at their binding sites (namely R95C, R116Q, R141C, R161C, R225C/L/H and R229W/Q). Some of these mutations can also produce a loss of PLP π-stacking interactions (mutations at Arg225) or inter-chain bonds (R116Q). The next category groups mutations affecting protein fold and stability due to non-conservative substitution, with impacts ranging from mild loss of stabilizing surface (E50K) and hydrophobic (P213S) interactions to destabilizing clashes (G118Q/R). Another category groups variants with a loss of residues (deletions, stop codons, frame shifts, non-sense mutations, etc.), disrupting the compact structure of the PNPOx homodimer and, consequently, the production of a functional protein. The last group includes an N-terminal extension that might mildly affect its potential unknown function.

Pathogenic mutations for which structural information is available, namely at Arg225, Arg229 and Arg116 in HsPNPOx, have also been biochemically studied. The R229W missense mutation was soon related to neonatal epileptic encephalopathy as a consequence of a drastic decrease in PNP oxidase activity and failure to maintain PLP levels, while the R229Q mutation produced moderate global developmental delay [24,52]. Seizures produced via the R229W mutation were resistant to PN but ceased after PLP treatment, and similarly, patients suffering from the R229Q mutation reduced seizures when treated with PLP [44,50]. R229W and R229Q mutations affect the last arginine in a highly conserved sequence—RLHDR—that contributes to binding FMN tightly to the active site (Figure 4). In vitro studies indicated that the R229W and R229Q mutations have no impact on overall structural integrity, including homodimer formation and conformational thermal stability. Nonetheless, their *k*_cat_ values resulted in 4.5- and 3.6-fold lower than those of WT, while *K*_M_^PNP^ increased by 192- and 129-fold, respectively [24]. In addition, both mutants showed a decrease in FMN affinity (*K*_d_^FMN^ being 50-fold and 3-fold higher than WT, respectively). Therefore, both pathogenic mutants, particularly R229W, lead to a significant decrease in catalytic efficiency (*k*_cat_/*K*_M_). As indicated above, the crystal structure of the R229W variant shows structural alterations in the active site (Figure 2d): loss of H-bond and salt-bridge interactions of FMN with Ser175 and Arg229, as well as the loss of critical H-bonds involving His227 and Arg225, which are important residues for substrate binding and orientation for catalysis. Unlike Trp, Gln has a similar size to Arg, suggesting that, in this case, steric interference may not be a factor for observable changes in oxidase activity. Loss of H-bonds between Ser175 and Glu217 with FMN could explain reduced affinity. While R229W lost FMN quite easily during purification, the remaining residues in the FMN binding pocket ensured its binding and catalytic orientation. Most likely, Gln229 in R229Q can still H-bond with FMN and does not alter significantly other interactions in the pocket.

In agreement with the above-indicated structural information, the conformational characterization of R116Q HsPNPOx suggests that the mutation does not alter overall structure integrity despite affecting the enzyme’s thermal stability [29]. This observation agrees with the mutation preventing one of the salt bridges that stabilizes the interface between both protomers in the homodimer. This has an impact on the enzyme’s affinity for FMN (*K*_d_^FMN^ 20-fold higher than WT), which sits at the interface between protomers, and as a consequence, on the catalytic efficiency that is reduced up to 40% of that of WT due to a decrease in *k*_cat_ and an increase in *K*_m_^PNP^. The R116Q mutation has been shown to hardly reduce PLP affinity, but it impairs the transfer of PLP to PLP-dependent enzymes, reducing the reactivation of the corresponding apo-enzymes [53,54]. Such impairments have also been observed in other mutations, such as R95C, and might have drastic effects on neurotransmitter biosynthesis.

Other HsPNPOx pathogenic variants responsible for PNPOD have also been more recently evaluated, namely G118R, R141C, R225H, R116Q/R225H and X262Q (replacement of the stop codon with a glutamine residue, leading to the formation of a 28-amino-acid-longer protein at the C terminal) [29]. Like the above-described mutations, these replacements also mainly negatively impact substrate and FMN cofactor binding and, as a consequence, the enzyme’s catalytic activity, but in general, they hardly impact the enzyme’s allosteric properties.

Besides PNPOD, the roles of PLP and HsPNPOx have been underscored in other diseases. Type 2 diabetes and chronic inflammation have been associated with decreased levels of PLP [55], and other studies relate PLP blood levels to the risk reduction of some cancers [56]. HsPNPOx has been reported to promote cell proliferation, migration and invasion, as well as to inhibit cancer cell apoptosis in breast cancer [57]. Despite being proposed as an oncogene, the mechanism of HsPNPOx participation in human cancers needs further study, as it may play different roles in certain cancer types: it is overexpressed in a large variety of cancers, such as breast, ovarian and colorectal cancer [57,58,59], but its expression appears decreased in others or has even been proposed to act as a protective factor [59]. Such evidence has suggested that the mechanism of HsPNPOx may be dual, combining regulation of the metabolism and interaction with signalling pathways. Therefore, although metabolic regulation may be common, differences in the regulation of signalling pathways may explain the heterogeneous behaviour of HsPNPOx in human cancers [59]. Finally, since HsPNPOx may play important roles during the development of cancer, it could serve as a potential tumour progression biomarker and, consequently, as a potential therapeutic target.

## 4. Functional and Structural Features of *Escherichia coli* PNPOx

The *E. coli* PNPOx (EcPNPOx) is one of the best-studied enzymes. In addition to participating in the PLP salvage pathway, such as HsPNPOx, it is the last enzyme in the de novo PLP biosynthesis pathway, being key in PLP homeostasis and bioavailability [21]. Unlike HsPNPOx, EcPNPOx greatly favours the transformation of PNP over PMP (*K*_m_^PMP^ is 50-fold higher than *K*_m_^PNP^) [32]. Nonetheless, despite its relevant function, EcPNPOx is also a poorly efficient enzyme (Table 1). EcPNPOx was soon shown to be controlled by inhibition of its PLP product, as well as to bind PLP tightly at a secondary non-catalytic site on each monomer, remaining bound during size-exclusion chromatography and purification but readily able to be transferred to PLP-client enzymes [60]. Thus, the EcPNPOx non-catalytic site was proposed to sequester PLP to channel it to client enzymes. More recently, mutagenesis, kinetic and equilibrium studies have demonstrated that PLP inhibition of EcPNPOx is actually of a mixed type resulting from product binding at the allosteric non-catalytic site, also pointing to structural and functional connections between the active and the allosteric sites [33]. Thus, EcPNPOx would suffer an allosteric feedback inhibition where the binding of the substrate at the active site and binding of PLP at the allosteric site negatively affect each other. This would produce a non-competent catalytic PNP–EcPNPOx–PLP ternary complex that reduces the ability of PLP to bind at the active site. Moreover, since binding of PLP at the non-catalytic allosteric site of free EcPNPOx has a higher affinity than binding at the active site, PLP will preferentially bind to the allosteric site in the free enzyme.

**Figure 5:**
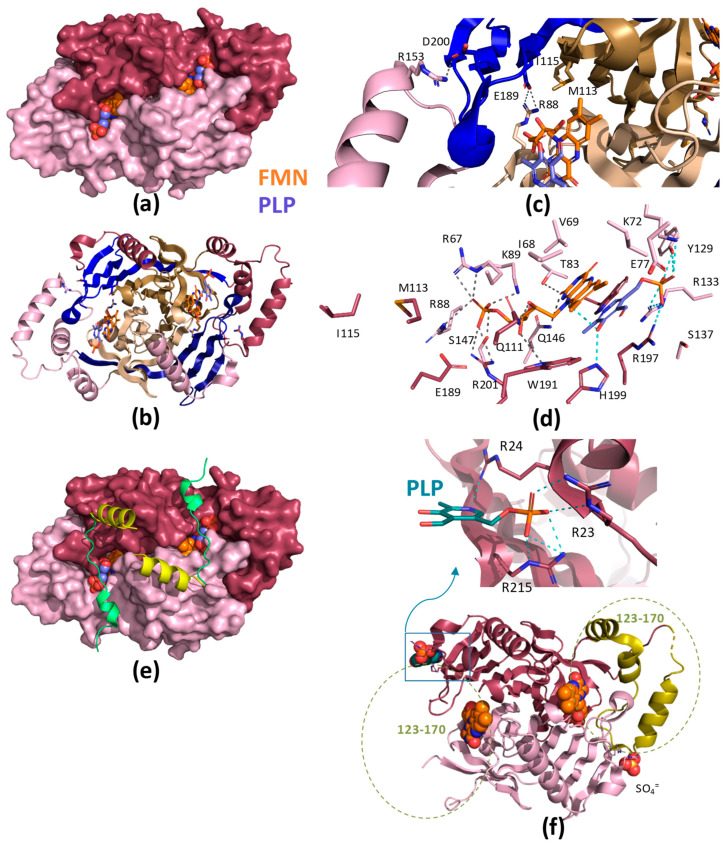
Structural features of EcPNPOx. (**a**) Surface representation of the crystallographic structure of WT EcPNPOx homodimer in complex with PLP at the active site (PDB 1G77). (**b**) Cartoon model of the homodimer, with the Putative_PNPOx domains in each protomer, highlighted in dark and light sand, respectively, and the corresponding PNP_phzG_C domains in light and dark blue. Residues involved in potential inter-subunit contacts are highlighted in the sticks. (**c**) Detail of inter-subunit contacts at the FMN of one active site. (**d**) Detail of the active site environment in the WT structure in complex with PLP. Polar contacts for FMN and PLP are shown as dashed lines in grey and cyan, respectively. Other colour codes as in panel (**a**). (**e**) N-terminal tail as observed in some structures in the active site environment. The position of the N-terminal is shown as a cartoon at each protomer in a PNPOx-free structure (PDB 6YLZ, yellow tail) and in a complex of PNPOx with PLP (PDB 1JNW, light green), while the rest of the structure is shown as surface. (**f**) Structure of a quadruple mutant at the active site of EcPNPOx with a PLP molecule bound at one of the allosteric sites and a sulphate ion at the other (PDB 1YMH). The inter-domain region observed only in one of the protomers is highlighted in pale green, and its position in both protomers is highlighted by a dashed circle of the same colour. In all panels, one protomer of the homodimer is shown in raspberry and the other in pale pink. FMN, PLP at the active site, and PLP at the allosteric site are shown in CPK, coloured in orange, violet or green teal spheres or sticks, respectively. Figure produced using PyMOL [39].

Several crystallographic structures are reported for EcPNPOx (Figure 5). This includes structures of WT both free (1WV4 and 1DNL) and in complex with PLP at the active site as well as at other different sites (1G76, 1G77, 1G78, 1G79 and 1JNW). Recently, structures of a mutant (K72I:Y129F:R133L:H199A), whose active site is impaired, have been reported both free and in complex with PLP at the non-catalytic allosteric site (6YLZ and 6YMH, respectively). Despite some structural variations, the overall folding and binding sites for the FMN and the catalytic substrate are highly conserved in comparison to HsPNPOx (Figure 5a,b). Hydrophobic interactions of FMN isoalloxazine with Val69A and Trp191B (Leu97A and Trp219B in HsPNPOx) are conserved. FMN also contributes to inter-subunit interactions, both direct and water-mediated, that are mostly conserved across species (Figure 5c). The main differences relate to Arg88 and Met113 (Arg116 and Arg141, respectively, in HsPNPOx). In EcPNPOx, Arg88 bridges both the phosphate moiety of FMN and the side chain of Glu189, while in HsPNPOx, the corresponding Arg116 participates in an inter-subunit salt bridge with Glu143 (Ile115 in EcPNPOx). On its side, Met113 in EcPNPOx does not support the stabilization of the phosphate group of FMN, contrary to the anchoring provided by Arg141 in HsPNPOx. In addition, the Arg153–Asp200 inter-subunit interaction appears more sluggish in the EcPNPOx structures than the corresponding Arg181–Asp228 one in HsPNPOx. Nonetheless, all residues at the PLP catalytic site remain strictly conserved in EcPNPOx compared to its human homologue (Figure 5d).

The available EcPNPOx crystal structures show different unit cells and active site conformations that range from open conformations in the absence of PLP to partially or closed conformations in their presence, as well as through conformations showing major disorder [26]. In general, PLP binding causes residues 129–140 and 193–199 to move towards the active site, allowing Tyr129, Arg133, Ser137, Arg197 and His199 to interact with PLP, making the active site partially but not completely closed (Figure 5d). The N-terminus appears quite flexible in general and is predicted as disordered, but some structures show that it might fold in an α-helix that stretches over the mouth of the active site to sequester bound PLP, with Arg14 and Tyr17 H-bonding it [21,26] (Figure 5e). Comparison of such crystal forms with data from mutational analyses allowed us to envisage a molecular mechanistic pathway for EcPNPOx [26]. It initiates with the binding of PNP at the active site of the resting enzyme. Subsequently, the catalytic stage produces PLP, and finally, the PLP product is transferred from the active site to the allosteric site for subsequent transfer to vitamin B_6_ apo-enzymes [28]. These studies also showed a relevant role for Arg197 (Figure 5d), both in binding and catalysis, with its guanidinium side chain being key in determining the enzyme stereospecificity and contributing to accepting the pair of electrons on C4′ of PNP that are transferred to FMN as a hydride ion [26].

Mutagenesis and structural analysis have located the allosteric PLP binding site in EcPNPOx at the interface between protomers, formed using an intricate electrostatic arginine cage comprised of Arg23, Arg24 and Arg215 (all within the same protomer), which effectively captures PLP through its phosphate group (Figure 5f) [21]. Manipulation of EcPNPOx allosteric properties has been achieved through site-directed mutagenesis targeting the arginine cage, unveiling a spectrum of effects ranging from the relaxation of allosteric coupling (observed in R23L/R24L and R23L/R215L variants) to the complete abolition of allosteric properties (as seen in R23L/R24L/R21L). Notably, the crystal structure of this EcPNPOx–PLP–non-catalytic complex exhibits large structural asymmetry. Specifically, only the allosteric arginine cage in one monomer tightly binds PLP, while helices 123–130, 135–143 and 153–166 in the other monomer (forming the interdomain cap that folds out of the PNPOx and PNP_phzG_C domains) undergo disorder upon PLP binding (Figure 5f). Such disorder is particularly localized to the surface cleft between the arginine cage of the first monomer and the N-terminal portion of helix 153–166 in the other monomer.

Altogether, these observations offered valuable insights into the allosteric regulation of EcPNPOx activity [21]. Thus, researchers in the field have shown that EcPNPOx is regulated via an allosteric inhibition mechanism that makes the EcPNPOx–PNP–PLP ternary complex catalytically incompetent. Such regulation occurs between two binding events, PNP and PLP, as a consequence of the structural perturbations induced on the protein by both upon binding. PLP binding at the Arg cage produces an important rearrangement at the active site, displacing some highly conserved residues regarding both the FMN and the PNP substrate and thus preventing the correct orientation of the substrate for stereospecific hydride transfer to FMN. Similarly, PNP binding at the active site is expected to impact PLP binding at the allosteric site, aligning with studies envisaging flexibility at the monomer inter-domain region and contributing to widening the channel for PLP passage between active and allosteric sites [28,44]. In this way, the enzyme’s function might be finely tuned through structural perturbations induced by the binding of both PNP and PLP. In addition, tight binding of PLP to the allosteric site in EcPNPOx will protect against the reactivity of its aldehyde group from the environment while allowing its concerted transfer to PLP-dependent apo-enzymes. Hence, this implies the involvement of EcPNPOx in facilitating the intracellular PLP delivery process.

## 5. Species-Specific Features in PNPOx

In addition to HsPNPOx and EcPNPOx, early studies reported kinetic and interaction parameters for PNPOx from various mammalian sources (Table 1), showing that, in general, PNPOx binds FMN with affinities in the nM range and is, at least in vitro, a relatively inefficient enzyme from a catalytic standpoint. PNPOx enzymes are characterized by sluggish *k*_cat_ values, with *K*_m_ and *K*_d_ values for substrates and the PLP product generally falling in the low micromolar range. The low *k*_cat_, together with the inhibition produced by the PLP substrate, hinders the comparison of values from different preparations or obtained under different assay conditions for the same PNPOx species (Table 1). Nonetheless, all of them are characterized by low *k*_cat_ and catalytic efficiencies, as well as by inhibition by the PLP product.

**Figure 6:**
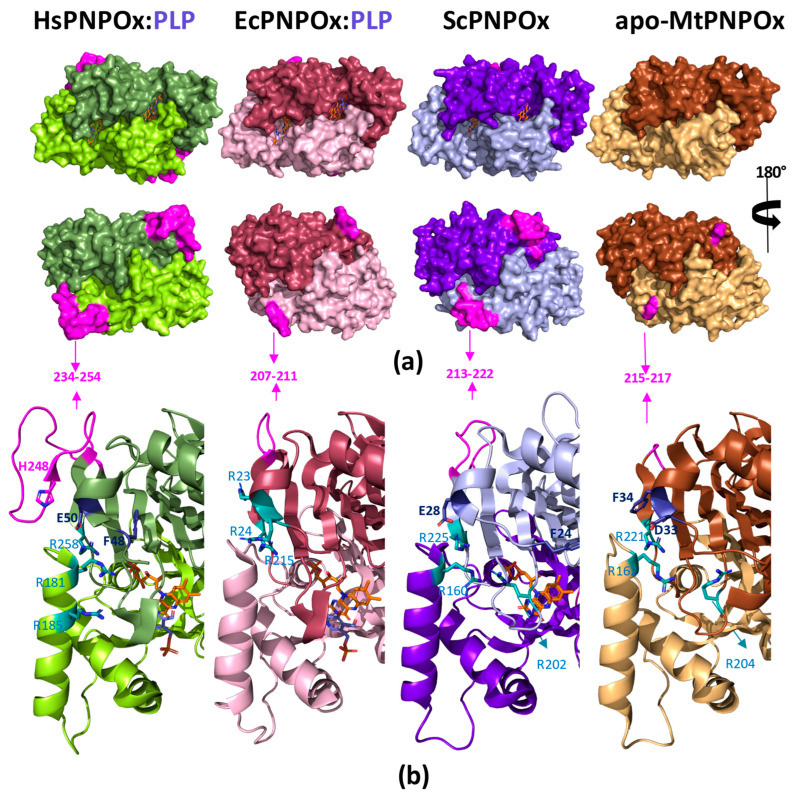
Species-specific structural features in PNPOx enzymes. (**a**) Surface representation of the crystallographic structure of the WT homodimers: from left to right, HsPNPOx in complex with PLP at the active site (1NRG), EcPNPOx in complex with PLP at the active site (PDB 1G76), ScPNPOx (1CI0) and MtPNPOx (2A2J). (**b**) Detail of differential structural features. Arg residues that might form Arg cages at positions close to that determined in EcPNPOx as the allosteric PLP binding site are highlighted in CPK, coloured sticks with carbons in deep teal. In HsPNPOx, in addition to Arg258, His248 (in CPK magenta sticks) at the loop connecting the C-terminal β-hairpin and Phe48 and E50 (in CPK dark blue sticks) at the N-terminus have been proposed to replace the Arg cage in allosteric PLP binding. Potential equivalent residues in ScPNPOx and MtPNPOx structures are similarly highlighted. In all panels, the loop connecting the C-terminal β-hairpin is highlighted in magenta, and each homodimer is shown in a different colour. FMN and PLP at the active site, when present in structural models, are shown in CPK sticks, coloured orange and violet, respectively. Figure produced using PyMOL [39].

Crystallographic structures are also available for PNPOx from the yeast *S. cerevisiae* (ScPNPOx, PDB 1CI0) and *Mycobacterium tuberculosis* (MtPNPOx, PDB 2A2J), but none of them has been functionally evaluated (Figure 6). Sequence identity within the assessed mammalian PNPOx states exceeds 90% (Table 2), but it decreases to around 40% when comparing mammalian enzymes to any of the others (Table 2). Moreover, pairwise sequence identity among ScPNPOx, EcPNPOx and MtPNPOx ranges by 40%, envisaging distinctive features among them.

Particularly worth noting are those at the N-terminus, where HsPNPOx is approximately 25 residues longer than EcPNPOx, MtPNPOx and ScPNPOx. Moreover, in HsPNPOx, the first 46–48 residues are predicted as disordered, and only a few of them are seen in some of its reported structures. A similar scenario generally occurs for the 19–25 N-terminal residues in the shorter homologues. In fact, this N-terminus hardly exhibits conservation among species (Figure 3 and Figure 4), with Tyr41 (number corresponding to HsPNPOx) being the only apparent conserved residue. This Tyr41, together with Asp44, replaces Arg14 and Tyr17 in EcPNPOx, which have been proposed to block access to the active site during the catalytic cycle (Figure 5e). Notably, in HsPNPOx, they were proposed as candidates to also H-bond to PLP, creating a lid over the active site, as the other residues would do in EcPNPOx [27]. Asp44 is also conserved in ScPNPOx and shows a conservative replacement in MtPNPOx. Additionally, EcPNPOx includes in this N-terminal region a part of the key motif (Arg23–Arg24) implicated in the PLP allosteric site that is absent at the N-terminus of the other evaluated enzymes. Interestingly, another significant insertion (15–20 residues) near the C-terminus of HsPNPOx (Figure 4) results in a considerably longer loop compared to ScPNPOx, EcPNPOx and particularly MtPNPOx (where it is almost non-existent) (Figure 6). This makes the structure of HsPNPOx unique at the loop connecting the C-terminal β-hairpin. This observation may be solely structural without a direct catalytic role, but the terminal β strand contains a highly conserved Arg, Arg258 in HsPNPOx, that corresponds to the third Arg cage residue (Arg215) identified in the PLP allosteric site in EcPNPOx. Notably, this Arg is also conserved in ScPNPOx and MtPNPOx (Figure 4 and Figure 6). Since allosteric regulation of PNPOx relies on biomolecular conformations with their corresponding dynamic transitions, such insertion introduces steric obstacles impeding HsPNPOx from conserving exactly the same PLP allosteric site as EcPNPOx. In addition, the absence of the Arg23–Arg24 motif also indicates that allosteric sites in ScPNPOx and MtPNPOx might not be similarly conserved regarding EcPNPOx.

Some HsPNPOx structures showed a phosphate bound close to the PLP allosteric site in EcPNPOx, particularly surrounded by residues His248 and Arg249 situated in the strand C-terminal β-hairpin strand, nearly at the edge of the inter-subunit contact interface. At a certain point, this site was proposed as potentially allocating the allosteric site. Such sites would not be conserved in ScPNPOx or MtPNPOx because their sequences are considerably shorter in this region. Nonetheless, the careful observation of these structures reveals other conserved patches of Arg residues that might form the cages to allocate PLP at the contact interface between both monomers in the homodimer. In HsPNPOx, such a patch might be formed by the highly conserved Arg258 of one protomer together with Arg181 and Arg185 situated in the other protomer in one of the helices that undergo disorder upon PLP binding at the allosteric site in EcPNPOx (Figure 6). Notably, molecular dynamics simulations in HsPNPOx identified the 181–195 α6 helix and the 195–198 β-turn at the interdomain cap folding out of the PNPOx and PNP_phzG_C domains as the most flexible region of the protein [31]. Arg181 at the N-terminus of the longest helix is conserved in ScPNPOx and MtPNPOx (as Arg160 and Arg162, respectively) but not Arg185. Nonetheless, in these two structures, the respective Arg202 and Arg204 residues, situated in the first protomer in a long loop that protrudes inside the other protomer surface, are ready to close the cage (Figure 6b). Nonetheless, more recent docking, mutational, and mechanistic studies indicate that in HsPNPOx, both the C-terminal β-hairpin and the N-terminus of the same protomer will generate a cleft for the binding of allosteric PLP, which will be stabilized via His248 at the loop and the highly conserved Arg258 at the end of the second strand of the C-terminal β-hairpin, together with the side chains of Phe48 and Glu50 at the flexible N-terminus (see Figure 1 in [30]). Here, it is worth noticing the diversity of conformations among the five available HsPNPOx structures at the first residues detected at the N-terminus (residues 46–52), which suggests flexibility in such structural element when PLP binds at the allosteric site (see Figure 6 in [30]).

Due to their shorter loops at the C-terminal β-hairpin, ScPNPOx and MtPNPOx lack a residue equivalent to His248 in HsPNPOx. However, these two proteins show their flexible N-terminus Phe and Glu/Asp residues that might contribute to the allosteric PLP binding, as suggested in HsPNPOx (Figure 6b). Therefore, despite in all evaluated PNPOxs, the N-terminal and C-terminal of the same protomer appear as the main contributors to allosteric PLP binding, it is clear that the different lengths in the loop connecting the C-terminal β-hairpin will induce species-specific features. In agreement, despite both HsPNPOx and EcPNPOx exhibiting PLP allosteric inhibition, the coupling between the allosteric and active sites appears notably weaker in HsPNPOx. In addition, while PLP does not bind at the active site of native EcPNPOx, HsPNPOx has demonstrated the capability to form a complex with two PLP molecules per protomer [21,27,29,33]. Such differences have been related to the binding of PLP at the allosteric site of EcPNPOx, which takes place with considerably higher affinity and might be behind the differing kinetic inhibition models that describe this enzyme in different species.

Structural analyses also find MtPNPOx to be the most divergent available experimental model. Notably, its crystal structure was reported in the absence of the FMN cofactor or any other ligand. Moreover, it lacks the arginine residue conserved as Arg88 in EcPNPOx, Arg166 in HsPNPOx and Arg95 in ScPNPOx. Nonetheless, despite the conservation of such Arg and Glu189 in all these species at the FMN binding site, the Arg88-Glu189 salt bridge present in EcPNPOx is not observed either in HsPNPOx or in ScPNPOx. Finally, it is also worth noting that the Cys residues at the monomers interface in HsPNPOx are absent in the other structures. All these features can, therefore, contribute to the intricate and reciprocal influence between PNP and PLP governing PNPOx activity in a species-specific manner.

## 6. PNPOx and Cofactor Channelling

PLP-client enzymes first undergo biosynthesis in the apo-form, subsequently transitioning into the catalytically active holo-PLP enzymes through the covalent binding of PLP to an active site lysine to form an aldimine [27]. This process is noteworthy due to the reactivity of PLP, which can potentially form aldimines with free amino groups on non-PLP proteins and, as a consequence, disrupt their function [10]. To mitigate this toxicity, the cell maintains a low concentration of free PLP through its dephosphorylation as well as via PLP feedback inhibition of PNPOx and PLK [33,61,62,63]. However, it is noteworthy how maintaining such a low concentration of free cellular PLP allows the activation of a large number of competing apo-PLP-dependent enzymes. A proposed protective mechanism would involve channelling the newly formed PLP using PNPOx exclusively to its client apo-PLP-dependent enzymes, offering additional advantages such as reduced transit time, mitigating loss through diffusion and avoiding competing metabolic pathways [61,64]. This hypothesis is supported by various biophysical and biochemical studies reporting the complex formation of PNPOx with different apo-PLP-client enzymes [65,66,67]. Likely due to their transient nature, experimental structures for such complexes have not been reported so far. As an alternative, computational approaches have served to simulate how HsPNPOx recognizes and interacts with its potential partners. Such an approach, in combination with experimental biochemical evaluations, was used to examine how HsPNPOx and PLP-dependent dopa decarboxylase (HsDDC) might recognize and interact with each other. The results suggested contact between the allosteric PLP site on HsPNPOx and the PLP active site of the receptor, as well as transient “loop-mediated” interactions between the two proteins while demonstrating PNPOx’s ability to both bind and transfer PLP (despite its tight binding) to apo-DDC [22].

Interestingly, the channelling approach in PNPOx may serve not only as a means to transfer its PLP product but also to integrate its own FMN cofactor, leading to the formation of the holo PNPOx state. Similarly to PLP, the FMN cofactor plays a crucial role in assisting the function of numerous flavoproteins and flavoenzymes within the cell (around 25% of flavoproteins use FMN in *Homo sapiens*) while maintaining its free concentration at a low level. FMN synthesis is catalysed by riboflavin kinase (RFK), and the transient interaction between RFK and PNPOx has been confirmed in the case of *H. sapiens* enzymes [31,68]. Furthermore, these studies have demonstrated the transfer of tightly bound FMN from HsRFK to apo-HsPNPOx, as well as the mutual influence between the two proteins in catalysis and ligand binding steps. Plausible binding modes of HsRFK binding to HsPNPOx have been simulated, indicating coupling between their respective FMN binding cavities. Various complex arrangements are envisaged to result from diverse ligation and conformational states expected for HsRFK during catalysis, particularly influenced by changes in the conformation of the loops forming the FMN binding site and its exit channel [68]. Importantly, predictions suggest that loops in HsPNPOx also contribute to protein–protein fitting, hinting again at transient “loop-mediated” interactions whose reorganization might mediate cofactor transfer [31]. Altogether, an intricate thermodynamic landscape governing binding and transference, and highly sensitive to particular HsRFK and HsPNPOx ligation states, was proposed to regulate coupling and overall channelling processes between these two proteins [31]. Residues predicted to be involved in this interaction in HsPNPOx were located in α1 (Phe66 and Glu67) and α4-tun (Glu152-Glu153, Glu155, Tyr157-Phe158 and Arg161-Pro162), and to a lesser extent in the β3-β4-hairpin (Phe102 and Arg108). Notably, some of these residues are conserved in EcPNPOx, ScPNPOx and MtPNPOx, especially those in α1 (Phe35 and Glu36 in EcPNPOx; and Phe43 and Glu45 in ScPNPOx) and α4-turn (Glu125, Tyr129-Phe130 and Arg133-Pro134 in EcPNPOx; Glu131, Glu134, Tyr136-Phe137 and Arg140-Pro141 in ScPNPOx; and Glu133-Glu134, Tyr138, Arg142-Pro143 in MtPNPOx).

## 7. Unlocking the Therapeutic Potential of PNPOx

Our current understanding, encompassing functional and structural aspects of PNPOx, focuses only on a few organisms. The studies on HsPNPOx and EcPNPOx have particularly provided insights into features governing their activity. Nonetheless, while they show shared characteristics, it is clear that PNPOx enzymes exhibit unique species-specific structural features tailored to regulate the allosteric modulation of their enzymatic activities, highlighting the adaptability of their mechanisms to meet the distinct needs of individual species. In the case of HsPNPOx, understanding the structural and mechanistic details behind its allosteric regulation and the implication on those processes of the dynamics of its homodimeric flavoenzyme architecture is crucial for advancing therapeutic strategies. This is of particular interest in the realm of conditions related to the metabolism of B_6_ vitamers, PNPOD, or even the progression of some tumours. In vivo, allosteric regulation plays a pivotal role in fine-tuning PNPOx activity in response to cellular conditions, being, therefore, a key target for modulators designed to selectively enhance or inhibit its function. Thus, it is clear that patients suffering from PNPOx mutations in different structural elements cannot all be treated in the same way. Thus, leveraging research into the PNPOx structure will allow medicinal practitioners to develop targeted therapies for particular conditions, ultimately offering new avenues for precision medicine approaches customized to individual patient needs. Structural information can facilitate the development of novel diagnostic tools that might use PNPOx as a potential biomarker for predicting patient prognosis, drug sensitivity and immunoreaction in some cancers. In addition, it might be of use for monitoring treatment responses for pathogenic mutations as well as for genetic polymorphisms and aiding in rational drug design, facilitating the optimization of pharmacokinetic properties and enhancing the efficacy and specificity of therapeutic interventions.

Further work must also be conducted in the functional and structural characterization of the molecular mechanisms of PNPOx in different organisms. Understanding the diversity of PNPOx regulation among species might also underscore its relevance for developing precision antimicrobial therapies that effectively target specific microbial pathogens while preserving the microbiota and minimizing adverse effects on human or animal health. In this context, structural insights into allosteric sites and conformational changes induced by the regulatory PLP molecule will enable the design of modulators that can selectively enhance or inhibit PNPOx activity. Moreover, the homodimeric nature of PNPOx also suggests potential avenues for therapeutic intervention in the development of antimicrobials, as disrupting protein–protein interactions within the dimer interface could alter enzyme function. Finally, continuing research in the field of PLP transfer and channelling to PLP-dependent enzymes is key because we have limited molecular knowledge of these processes. A deeper understanding of such mechanisms will surely lead to insights into the regulation of metabolic pathways and the mechanisms underlying diseases associated with PLP-dependent enzyme dysfunction. Uncovering the intricate details of such processes will help to identify potential targets for therapeutic interventions and develop novel treatments for vitamin B_6_ deficiency disorders, epilepsy and certain types of cancer. The precise coordination required for PLP delivery to specific enzymes highlights the complexity of cellular regulation and the interconnectedness of metabolic pathways. Unravelling these intricacies can provide valuable insights into cellular function and dysfunction, with implications for various fields, including biochemistry, pharmacology and medicine.

Looking ahead, future investigations into PNPOx may delve deeper into understanding its complex molecular mechanisms, uncovering additional allosteric sites, exploring interactions with other biomolecules and client enzymes, and elucidating its role in diverse physiological processes. In conclusion, by exploring the intricacies of PNPOx and PLP metabolism, researchers can pave the way for novel discoveries and applications that will benefit human health and technology.

## Figures and Tables

**Table 1:** Summary of kinetic and interaction parameters reported for PNPOx enzymes from different species. See Figure A1 for the illustration of the designations of constants.

Species	Conditions	*k*_cat_^PNP^ (s^−1^)	*K*_M_^PNP^ (µM)	*k*_cat_/*K*_M_^PNP^ (µM^−1^ s^−1^)	*k*_cat_^PMP^ (s^−1^)	*K*_M_^PMP^ (µM)	*k*_cat_*/K*_M_^PMP^ (µM^−1^ s^−1^)	*K*_I_^PLP^ (µM)	*K*_I_^PNP^ (µM)	*K*_d_^PLP^ (µM)	*K*_d_^FMN^ (nM)	Refs.
*H. sapiens*	50 mM Tris/HCl pH 7.6 37° ^1^	0.20 ± 0.01	1.8	0.11	0.20	1.0	0.2	3.2				[27]
	50 mM Tris/BES pH 7.6 37°	0.20 ± 0.01	2.4 ± 0.2	0.08 ± 0.01							14 ± 2 ^2^	[24]
	50 mM Tris/HCl, 5 mM β-mercaptoeth. pH 7.6 37°	0.06 ± 0.01	2.6 ± 0.2	0.02 ± 0.01	0.13 ± 0.01	61 ± 6	0.002 ± 0.001	0.6 ± 0.1		1.0 ± 0.1 3.2 ± 0.2	13 ± 2	[29,30]
	50 mM HEPES pH 7.6 37°	0.06 ± 0.01	2.8 ± 0.2	0.02 ± 0.01				0.9 ± 0.1				[29]
	50 mM Tris/HCl pH 7.6 37°	0.20 ± 0.01	2.4 ± 0.2	0.08 ± 0.01								[31]
*E. coli*	50 mM Tris pH 7.6 37°	0.3	2	0.15								[25]
	NaPi/HEPES pH 7.6 37°	0.2	2	0.1								[25]
	200 mM Tris-HCl, 200 mM KPi pH 8.5 37°	0.8	2	0.4	1.7	105	0.02	8				[32]
	50 mM Na/HEPES pH 7.6	0.25 ± 0.01	1.6 ± 0.4	0.02 ± 0.01				0.28 ± 0.01		0.15		[33]
Sheep brain	100 mM KPi pH 8.0 25°	0.2	13	0.01				4				[34]
	100 mM KPi pH 7.4 25°							2				[35]
Rabbit liver	200 mM Tris pH 8.0 37°		30			10					40	[36]
	200 mMTris/HCl pH 8.0 25°	0.7	8.2	0.1	0.1	3.6	0.03		50	45		[37]
Pig brain	100 mM PPi pH 8.4 25°	0.09	13	0.007	0.009	100	0.00009					[38]

^1^ All temperatures are given in °C. ^2^ NaPi pH 7.2 25°.

**Table 2:** Percentage of sequence identity among PNPOx enzymes from different species structurally or functionally characterized.

Species	HsPNPOx	EcPNPOx	MtPNPOx	ScPNPOx	Rabbit	Pig	Sheep
HsPNPOx	100						
EcPNPOx	43.8	100					
MtPNPOx	38.1	41.5	100				
ScPNPOx	38.1	41.6	37.8	100			
Rabbit	91.2	40.5	38.4	39.7	100		
Pig	89.7	41.5	38.0	38.0	87.0	100	
Sheep	89.7	43.0	42.5	41.2	87.0	88.9	100

**Figure A1:**
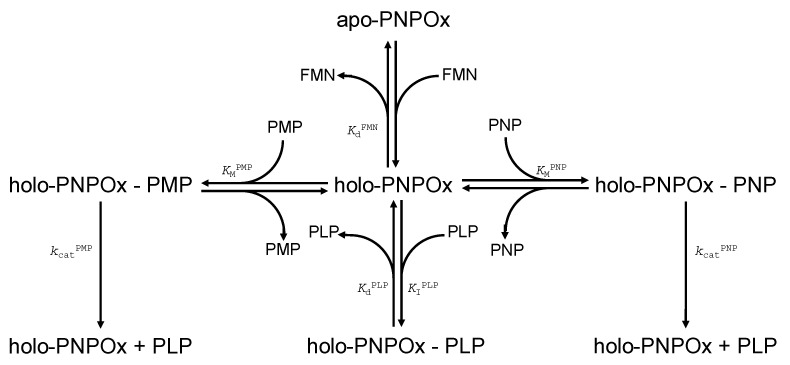
Catalytic scheme illustrating the designations of kinetic and equilibrium constants in Table 1.

## Data Availability

The data presented in this study derived from public databases, namely PDB [https://www.rcsb.org/] and public bibliographic databases such as PubMed [https://pubmed.ncbi.nlm.nih.gov/] as revised up to 15th February 2024.

## Appendix A

Abbreviations: pyridoxine (PN), pyridoxal (PL), pyridoxamine (PM), pyridoxamine-5′-phosphate (PNP), pyridoxal-5′-phosphate (PLP), pyridoxamine-5′-phosphate (PMP), pyridoxal kinase (PLK), pyridox-(am)-ine 5′-phosphate oxidase (PNPOx), *Homo sapiens* PNPOx (HsPNPOx), *Escherichia coli* PNPOx (EcPNPOx), *Saccharomyces cerevisiae* PNPOx, *Mycobacterium tuberculosis* PNPOx (MtPNPOx).

## Appendix B

Bibliographic searches were pursued by cross-examining the Protein Data Bank, the Online Mendelian Inheritance in Man Database (OMIM) and the PubMed Database. Interrogating terms were PLP, PNPOx, and/or PNPOD. The retrieved manuscripts were further searched for a bibliography that was not found in those databases. More than 120 papers were searched, referencing here those that were more in accordance with the presented information.

## References

[1] Wilson M.P., Plecko B., Mills P.B., Clayton P.T. (2019). Disorders affecting vitamin B metabolism. J. Inherit. Metab. Dis..

[2] Parra M., Stahl S., Hellmann H. (2018). Vitamin B_6_ and Its Role in Cell Metabolism and Physiology. Cells.

[3] Guerriero R.M., Patel A.A., Walsh B., Baumer F.M., Shah A.S., Peters J.M., Rodan L.H., Agrawal P.B., Pearl P.L., Takeoka M. (2017). Systemic Manifestations in Pyridox(am)ine 5′-Phosphate Oxidase Deficiency. Pediatr. Neurol..

[4] Percudani R., Peracchi A. (2003). A genomic overview of pyridoxal-phosphate-dependent enzymes. EMBO Rep..

[5] Percudani R., Peracchi A. (2009). The B6 database: A tool for the description and classification of vitamin B6-dependent enzymatic activities and of the corresponding protein families. BMC Bioinform..

[6] Gachon F., Fonjallaz P., Damiola F., Gos P., Kodama T., Zakany J., Duboule D., Petit B., Tafti M., Schibler U. (2004). The loss of circadian PAR bZip transcription factors results in epilepsy. Genes Dev..

[7] Laber B., Maurer W., Scharf S., Stepusin K., Schmidt F.S. (1999). Vitamin B6 biosynthesis: Formation of pyridoxine 5′-phosphate from 4-(phosphohydroxy)-L-threonine and 1-deoxy-D-xylulose-5-phosphate by PdxA and PdxJ protein. FEBS Lett..

[8] Tramonti A., Nardella C., di Salvo M.L., Barile A., D’Alessio F., de Crécy-Lagard V., Contestabile R. (2021). Knowns and Unknowns of Vitamin B. EcoSal Plus.

[9] Tambasco-Studart M., Titiz O., Raschle T., Forster G., Amrhein N., Fitzpatrick T.B. (2005). Vitamin B6 biosynthesis in higher plants. Proc. Natl. Acad. Sci. USA.

[10] di Salvo M.L., Safo M.K., Contestabile R. (2012). Biomedical aspects of pyridoxal 5′-phosphate availability. Front. Biosci. (Elite Ed.).

[11] Said H.M. (2011). Intestinal absorption of water-soluble vitamins in health and disease. Biochem. J..

[12] Shi X., Gao B., Srivastava A., Izzi Z., Abdalla Y., Shen W., Raj D. (2022). Alterations of gut microbial pathways and virulence factors in hemodialysis patients. Front. Cell. Infect. Microbiol..

[13] Denise R., Babor J., Gerlt J.A., de Crécy-Lagard V. (2023). Pyridoxal 5′-phosphate synthesis and salvage in Bacteria and Archaea: Predicting pathway variant distributions and holes. Microb. Genom..

[14] Gregory J.F. (1998). Nutritional Properties and significance of vitamin glycosides. Annu. Rev. Nutr..

[15] Said Z.M., Subramanian V.S., Vaziri N.D., Said H.M. (2008). Pyridoxine uptake by colonocytes: A specific and regulated carrier-mediated process. Am. J. Physiol. Cell Physiol..

[16] Suvorova I.A., Rodionov D.A. (2016). Comparative genomics of pyridoxal 5′-phosphate-dependent transcription factor regulons in. Microb. Genom..

[17] Said H.M., Ortiz A., Ma T.Y. (2003). A carrier-mediated mechanism for pyridoxine uptake by human intestinal epithelial Caco-2 cells: Regulation by a PKA-mediated pathway. Am. J. Physiol. Cell Physiol..

[18] Miyake K., Yasujima T., Takahashi S., Yamashiro T., Yuasa H. (2022). Identification of the amino acid residues involved in the species-dependent differences in the pyridoxine transport function of SLC19A3. J. Biol. Chem..

[19] Stolz J., Vielreicher M. (2003). Tpn1p, the plasma membrane vitamin B6 transporter of Saccharomyces cerevisiae. J. Biol. Chem..

[20] Ciapaite J., van Roermund C.W.T., Bosma M., Gerrits J., Houten S.M., IJlst L., Waterham H.R., van Karnebeek C.D.M., Wanders R.J.A., Zwartkruis F.J.T. (2023). Maintenance of cellular vitamin B6 levels and mitochondrial oxidative function depend on pyridoxal 5′-phosphate homeostasis protein. J. Biol. Chem..

[21] Barile A., Battista T., Fiorillo A., di Salvo M.L., Malatesta F., Tramonti A., Ilari A., Contestabile R. (2021). Identification and characterization of the pyridoxal 5′-phosphate allosteric site in Escherichia coli pyridoxine 5′-phosphate oxidase. J. Biol. Chem..

[22] Al Mughram M.H., Ghatge M.S., Kellogg G.E., Safo M.K. (2022). Elucidating the Interaction between Pyridoxine 5′-Phosphate Oxidase and Dopa Decarboxylase: Activation of B6-Dependent Enzyme. Int. J. Mol. Sci..

[23] Kang J.H., Hong M.L., Kim D.W., Park J., Kang T.C., Won M.H., Baek N.I., Moon B.J., Choi S.Y., Kwon O.S. (2004). Genomic organization, tissue distribution and deletion mutation of human pyridoxine 5′-phosphate oxidase. Eur. J. Biochem..

[24] Musayev F.N., Di Salvo M.L., Saavedra M.A., Contestabile R., Ghatge M.S., Haynes A., Schirch V., Safo M.K. (2009). Molecular basis of reduced pyridoxine 5′-phosphate oxidase catalytic activity in neonatal epileptic encephalopathy disorder. J. Biol. Chem..

[25] Di Salvo M., Yang E., Zhao G., Winkler M.E., Schirch V. (1998). Expression, purification, and characterization of recombinant Escherichia coli pyridoxine 5′-phosphate oxidase. Protein Expr. Purif..

[26] di Salvo M.L., Ko T.P., Musayev F.N., Raboni S., Schirch V., Safo M.K. (2002). Active site structure and stereospecificity of Escherichia coli pyridoxine-5′-phosphate oxidase. J. Mol. Biol..

[27] Musayev F.N., Di Salvo M.L., Ko T.P., Schirch V., Safo M.K. (2003). Structure and properties of recombinant human pyridoxine 5′-phosphate oxidase. Protein Sci..

[28] Safo M.K., Musayev F.N., Schirch V. (2005). Structure of Escherichia coli pyridoxine 5′-phosphate oxidase in a tetragonal crystal form: Insights into the mechanistic pathway of the enzyme. Acta Crystallogr. D Biol. Crystallogr..

[29] Barile A., Nogués I., di Salvo M.L., Bunik V., Contestabile R., Tramonti A. (2020). Molecular characterization of pyridoxine 5′-phosphate oxidase and its pathogenic forms associated with neonatal epileptic encephalopathy. Sci. Rep..

[30] Barile A., Graziani C., Antonelli L., Parroni A., Fiorillo A., di Salvo M.L., Ilari A., Giorgi A., Rosignoli S., Paiardini A. (2024). Identification of the pyridoxal 5′-phosphate allosteric site in human pyridox(am)ine 5′-phosphate oxidase. Protein Sci..

[31] Rivero M., Boneta S., Novo N., Velázquez-Campoy A., Polo V., Medina M. (2023). Riboflavin kinase and pyridoxine 5′-phosphate oxidase complex formation envisages transient interactions for FMN cofactor delivery. Front. Mol. Biosci..

[32] Zhao G., Winkler M.E. (1995). Kinetic limitation and cellular amount of pyridoxine (pyridoxamine) 5′-phosphate oxidase of Escherichia coli K-12. J. Bacteriol..

[33] Barile A., Tramonti A., di Salvo M.L., Nogués I., Nardella C., Malatesta F., Contestabile R. (2019). Allosteric feedback inhibition of pyridoxine 5′-phosphate oxidase from. J. Biol. Chem..

[34] Kim Y.T., Churchich J.E. (1989). Sequence of the cysteinyl-containing peptides of 4-aminobutyrate aminotransferase. Identification of sulfhydryl residues involved in intersubunit linkage. Eur. J. Biochem..

[35] Choi S.Y., Churchich J.E., Zaiden E., Kwok F. (1987). Brain pyridoxine-5-phosphate oxidase. Modulation of its catalytic activity by reaction with pyridoxal 5-phosphate and analogs. J. Biol. Chem..

[36] Kazarinoff M.N., McCormick D.B. (1975). Rabbit liver pyridoxamine (pyridoxine) 5′-phosphate oxidase. Purification and properties. J. Biol. Chem..

[37] Choi J.D., Bowers-Komro M., Davis M.D., Edmondson D.E., McCormick D.B. (1983). Kinetic properties of pyridoxamine (pyridoxine)-5′-phosphate oxidase from rabbit liver. J. Biol. Chem..

[38] Churchich J.E. (1984). Brain pyridoxine-5-phosphate oxidase. A dimeric enzyme containing one FMN site. Eur. J. Biochem..

[39] Delano W.L. (2002). PyMOL: An open-source molecular graphics tool. CCP4 Newsl. Protein Crystallogr..

[40] Ghatge M.S., Al Mughram M., Omar A.M., Safo M.K. (2021). Inborn errors in the vitamin B6 salvage enzymes associated with neonatal epileptic encephalopathy and other pathologies. Biochimie.

[41] Barile A., Mills P., di Salvo M.L., Graziani C., Bunik V., Clayton P., Contestabile R., Tramonti A. (2021). Characterization of Novel Pathogenic Variants Causing Pyridox(am)ine 5′-Phosphate Oxidase-Dependent Epilepsy. Int. J. Mol. Sci..

[42] Alghamdi M., Bashiri F.A., Abdelhakim M., Adly N., Jamjoom D.Z., Sumaily K.M., Alghanem B., Arold S.T. (2021). Phenotypic and molecular spectrum of pyridoxamine-5′-phosphate oxidase deficiency: A scoping review of 87 cases of pyridoxamine-5′-phosphate oxidase deficiency. Clin. Genet..

[43] Guerin A., Aziz A.S., Mutch C., Lewis J., Go C.Y., Mercimek-Mahmutoglu S. (2015). Pyridox(am)ine-5-Phosphate Oxidase Deficiency Treatable Cause of Neonatal Epileptic Encephalopathy with Burst Suppression: Case Report and Review of the Literature. J. Child. Neurol..

[44] Mills P.B., Camuzeaux S.S., Footitt E.J., Mills K.A., Gissen P., Fisher L., Das K.B., Varadkar S.M., Zuberi S., McWilliam R. (2014). Epilepsy due to PNPO mutations: Genotype, environment and treatment affect presentation and outcome. Brain.

[45] Khayat M., Korman S.H., Frankel P., Weintraub Z., Hershckowitz S., Sheffer V.F., Elisha M.B., Wevers R.A., Falik-Zaccai T.C. (2008). PNPO deficiency: An under diagnosed inborn error of pyridoxine metabolism. Mol. Genet. Metab..

[46] Plecko B., Paul K., Mills P., Clayton P., Paschke E., Maier O., Hasselmann O., Schmiedel G., Kanz S., Connolly M. (2014). Pyridoxine responsiveness in novel mutations of the PNPO gene. Neurology.

[47] Jaeger B., Abeling N.G., Salomons G.S., Struys E.A., Simas-Mendes M., Geukers V.G., Poll-The B.T. (2016). Pyridoxine responsive epilepsy caused by a novel homozygous PNPO mutation. Mol. Genet. Metab. Rep..

[48] Hoffmann G.F., Schmitt B., Windfuhr M., Wagner N., Strehl H., Bagci S., Franz A.R., Mills P.B., Clayton P.T., Baumgartner M.R. (2007). Pyridoxal 5′-phosphate may be curative in early-onset epileptic encephalopathy. J. Inherit. Metab. Dis..

[49] Chi W., Iyengar A.S.R., Fu W., Liu W., Berg A.E., Wu C.F., Zhuang X. (2022). *Drosophila* carrying epilepsy-associated variants in the vitamin B6 metabolism gene *PNPO* display allele- and diet-dependent phenotypes. Proc. Natl. Acad. Sci. USA.

[50] Ware T.L., Earl J., Salomons G.S., Struys E.A., Peters H.L., Howell K.B., Pitt J.J., Freeman J.L. (2014). Typical and atypical phenotypes of PNPO deficiency with elevated CSF and plasma pyridoxamine on treatment. Dev. Med. Child. Neurol..

[51] Farmania R., Gupta A., Ankur K., Chetry S., Sharma S. (2021). Complexities of pyridoxine response in PNPO deficiency. Epilepsy Behav. Rep..

[52] Mills P.B., Surtees R.A., Champion M.P., Beesley C.E., Dalton N., Scambler P.J., Heales S.J., Briddon A., Scheimberg I., Hoffmann G.F. (2005). Neonatal epileptic encephalopathy caused by mutations in the PNPO gene encoding pyridox(am)ine 5′-phosphate oxidase. Hum. Mol. Genet..

[53] di Salvo M.L., Mastrangelo M., Nogués I., Tolve M., Paiardini A., Carducci C., Mei D., Montomoli M., Tramonti A., Guerrini R. (2017). Biochemical data from the characterization of a new pathogenic mutation of human pyridoxine-5′-phosphate oxidase (PNPO). Data Brief..

[54] di Salvo M.L., Mastrangelo M., Nogués I., Tolve M., Paiardini A., Carducci C., Mei D., Montomoli M., Tramonti A., Guerrini R. (2017). Pyridoxine-5′-phosphate oxidase (Pnpo) deficiency: Clinical and biochemical alterations associated with the C.347g>A (P.·Arg116gln) mutation. Mol. Genet. Metab..

[55] Stach K., Stach W., Augoff K. (2021). Vitamin B6 in Health and Disease. Nutrients.

[56] Mocellin S., Briarava M., Pilati P. (2017). Vitamin B6 and Cancer Risk: A Field Synopsis and Meta-Analysis. J. Natl. Cancer Inst..

[57] Ren W., Guan W., Zhang J., Wang F., Xu G. (2019). Pyridoxine 5′-phosphate oxidase is correlated with human breast invasive ductal carcinoma development. Aging.

[58] Chen H., Sun X., Ge W., Qian Y., Bai R., Zheng S. (2017). A seven-gene signature predicts overall survival of patients with colorectal cancer. Oncotarget.

[59] Zhang L., Li X., Zhang J., Xu G. (2021). Prognostic Implication and Oncogenic Role of PNPO in Pan-Cancer. Front. Cell Dev. Biol..

[60] Yang E.S., Schirch V. (2000). Tight binding of pyridoxal 5′-phosphate to recombinant Escherichia coli pyridoxine 5′-phosphate oxidase. Arch. Biochem. Biophys..

[61] di Salvo M.L., Contestabile R., Safo M.K. (2011). Vitamin B(6) salvage enzymes: Mechanism, structure and regulation. Biochim. Biophys. Acta.

[62] Whittaker J.W. (2016). Intracellular trafficking of the pyridoxal cofactor. Implications for health and metabolic disease. Arch. Biochem. Biophys..

[63] Ghatge M.S., Contestabile R., di Salvo M.L., Desai J.V., Gandhi A.K., Camara C.M., Florio R., González I.N., Parroni A., Schirch V. (2012). Pyridoxal 5′-phosphate is a slow tight binding inhibitor of E. coli pyridoxal kinase. PLoS ONE.

[64] Miles E.W., Rhee S., Davies D.R. (1999). The molecular basis of substrate channeling. J. Biol. Chem..

[65] Ghatge M.S., Karve S.S., David T.M., Ahmed M.H., Musayev F.N., Cunningham K., Schirch V., Safo M.K. (2016). Inactive mutants of human pyridoxine 5′-phosphate oxidase: A possible role for a noncatalytic pyridoxal 5′-phosphate tight binding site. FEBS Open Bio.

[66] Kim Y.T., Kwok F., Churchich J.E. (1988). Interactions of pyridoxal kinase and aspartate aminotransferase emission anisotropy and compartmentation studies. J. Biol. Chem..

[67] Cheung P.Y., Fong C.C., Ng K.T., Lam W.C., Leung Y.C., Tsang C.W., Yang M., Wong M.S. (2003). Interaction between pyridoxal kinase and pyridoxal-5-phosphate-dependent enzymes. J. Biochem..

[68] Anoz-Carbonell E., Rivero M., Polo V., Velázquez-Campoy A., Medina M. (2020). Human riboflavin kinase: Species-specific traits in the biosynthesis of the FMN cofactor. FASEB J..

